# Change in hepatitis C virus positivity among needle-stick injury source patients: a 10-year experience in a Japanese tertiary hospital

**DOI:** 10.1186/s12879-021-06117-4

**Published:** 2021-04-30

**Authors:** Kazuya Okushin, Rie Suzuki, Takeya Tsutsumi, Koh Okamoto, Kazuhiko Ikeuchi, Akira Kado, Chihiro Minatsuki, Yuka Minami-Kobayashi, Nobuhiko Satoh, Mahoko Ikeda, Sohei Harada, Kenichiro Enooku, Hidetaka Fujinaga, Hiroshi Yotsuyanagi, Kazuhiko Koike, Kyoji Moriya

**Affiliations:** 1 Department of Infection Control and Prevention, Graduate School of Medicine, The University of Tokyo, 7-3-1 Hongo, Bunkyo-ku, Tokyo, 113-8655 Japan; 2 Department of Gastroenterology, Graduate School of Medicine, The University of Tokyo, Tokyo, Japan; 3 Division of Infectious Diseases, Advanced Clinical Research Center, The Institute of Medical Science, The University of Tokyo, Tokyo, Japan; 4 Department of Infectious Diseases, Graduate School of Medicine, The University of Tokyo, Tokyo, Japan

**Keywords:** Needle-stick injury, HCV, HCV antibody, HCV RNA

## Abstract

**Background:**

As a blood-borne pathogen, hepatitis C virus (HCV) has long been a major threat associated with needle-stick injuries (NSIs) mainly because no vaccine is available for HCV. Following an NSI, we usually test the source patient for HCV antibody (HCV-Ab). Since HCV-Ab positivity does not necessarily indicate current infection, HCV RNA is further examined in patients positive for HCV-Ab. Direct-acting antivirals (DAAs) have enabled us to treat most HCV-infected patients; therefore, we speculate that the rate of HCV RNA positivity among HCV-Ab-positive patients decreased after the emergence of DAAs. This cross-sectional study was performed to investigate the change in the actual HCV RNA positivity rate in source patients before and after the interferon (IFN)-free DAA era.

**Methods:**

This was a cross-sectional study of NSI source patients at a tertiary academic hospital in Japan from 2009 to 2019. IFN-free DAA regimens were first introduced in Japan in 2014. Accordingly, we compared HCV status of NSI source patients that occurred between 2009 and 2014 (the era before IFN-free DAAs) with those that occurred between 2015 and 2019 (the era of IFN-free DAAs) in a tertiary care hospital in Japan.

**Results:**

In total, 1435 NSIs occurred, and 150 HCV-Ab-positive patients were analyzed. The proportion of HCV RNA-positive patients significantly changed from 2009 through 2019 (*p* = 0.005, Cochran–Armitage test). Between 2009 and 2014, 102 source patients were HCV-Ab-positive, 78 of whom were also positive for HCV RNA (76.5%; 95%CI, 67.4–83.6%). Between 2015 and 2019, 48 patients were HCV-Ab-positive, 23 of whom were also positive for HCV RNA (47.9%; 95%CI, 34.5–61.7%; *p* = 0.0007 compared with 2009–2014). In the era of IFN-free DAAs, 9 of 23 HCV RNA-negative patients (39.1%) and 2 of 22 HCV RNA-positive patients (9.1%) were treated with an IFN-free combination of DAAs (*p* = 0.0351). Regarding the departments where NSIs occurred, HCV RNA-negative patients were predominant in departments not related to liver diseases in the era of IFN-free DAAs (*p* = 0.0078, compared with 2009–2014).

**Conclusions:**

Actual HCV RNA positivity in source patients of NSIs decreased after the emergence of IFN-free DAAs. IFN-free DAAs might have contributed to this reduction, and HCV RNA-negative patients were predominant in departments not related to liver diseases in the era of IFN-free DAAs.

**Supplementary Information:**

The online version contains supplementary material available at 10.1186/s12879-021-06117-4.

## Introduction

Healthcare workers (HCWs) are at risk of infection due to needle-stick injuries (NSIs) worldwide [1, 2]. Among the three major blood-borne pathogens transmitted through NSIs, i.e., hepatitis C virus (HCV), hepatitis B virus (HBV), and human immunodeficiency virus (HIV), immunization is available for only HBV (4). Moreover, postexposure management has been well established for HIV and HBV [3, 4]. In contrast, no vaccine is available for HCV [5]. Postexposure prophylaxis (PEP) strategies for HCV using direct-acting antivirals (DAAs) have been reported but are not considered cost-effective [6]. When HCWs suffer NSIs associated with an HCV-positive source patient, they can be only monitored for the development of hepatitis C. Consequently, HCV has long been a major threat in the context of NSIs despite its low rate of transmission (approximately 1.8%) due to the absence of valid immunization [7, 8].

When an NSI occurs, a source patient is tested for the presence of HCV antibody (HCV-Ab) [7, 9]. A positive HCV-Ab result indicates current or previous infection or a false-positive result [10]. The presence of serum HCV RNA indicates active HCV infection, and high HCV RNA levels appear to correlate with an increased risk of transmission [11, 12]. Consequently, HCV RNA is detected to confirm the current infection status in positive HCV-Ab patients [7, 9].

The reported prevalence of HCV among NSI source patients varies considerably depending on the patient population, geographical location and time period examined. For example, in Japan, the national burden of HCV infection was estimated in 2005. Tanaka et al. reported that 807,903 (95% CI 679,886–974,292) of the total population of 127,285,653 were estimated to be infected with HCV at a carrier rate of 0.63% [13]. Furthermore, in Japan, most hepatitis C positive patients are elderly [13] because the illicit intravenous amphetamine abuse during the turmoil period just after World War II is considered as a possible transmission route of HCV [14], in addition to current illegal injection drug use and previous transfusion of contaminated blood products which are common in other countries.

According to the prevalence of HCV among NSI source patients in Japan, Mitsui et al. reported that 91 patients (57.2%) were positive for HCV-Ab among 159 NSI source patients, and 81 patients (89.0%) were also positive for HCV RNA among 91 HCV-Ab-positive patients in 1992 [12]. However, since then, the actual HCV positivity rate among NSI source patients based on HCV RNA analysis has not been reported.

In the last decade, several interferon (IFN)-free DAAs have been developed and widely used for the treatment of chronic HCV infection. As IFN-free DAAs efficiently eliminate HCV and almost all patients achieve a sustained virological response (SVR) [15, 16], we speculated that HCV RNA positivity decreased after the introduction of IFN-free DAAs.

In this cross-sectional study, we retrospectively analyzed the HCV-Ab and HCV RNA statuses of NSI source patients to investigate the change in the HCV RNA positivity rate between the eras before and after the emergence of IFN-free DAAs.

## Materials and methods

### Patients

We retrospectively analyzed the laboratory data of NSI source patients at The University of Tokyo Hospital (UTH), Tokyo, Japan (1228 beds and approximately 4000 employees) from January 2009 to December 2019. UTH is a tertiary academic hospital that provides general and specialized services, including hepatology and liver transplantation. At UTH, all HCWs are required to report NSIs to the Department of Infection Control and Prevention according to the institutional guideline mandate. The reported data include date, site, NSI device, and the source patient’s blood-borne pathogen status. NSI cases were identified using the Department of Infection Control and Prevention database. We included all HCW NSI cases with HCV-Ab-positive source patients. We excluded cases with incomplete data, such as cases with incorrect medical record numbers for source patients or NSIs that occurred during forensic autopsy. Only the first NSI was analyzed if the same patient was the source in multiple NSI cases. We further excluded cases whose HCV RNA data were not available.

We manually extracted the following variables from the database and medical records of source patients: date, age, sex, HCV RNA status, HCV-Ab titer, and the department where the NSI occurred. We further extracted source patients’ histories of antiviral treatment for HCV prior to the incidence of NSIs. We dichotomized the study period into before interferon (IFN)-free DAAs (December 2014 and earlier) and after IFN-free DAAs (January 2015 and later) because the first IFN-free DAA regimen (daclatasvir hydrochloride and asunaprevir, DCV/ASV) was introduced in September 2014, followed by several IFN-free DAA regimens, such as ledipasvir/sofosbuvir (LDV/SOF), sofosbuvir/ribavirin (SOF/RBV), glecaprevir/pibrentasvir, and sofosbuvir/velpatasvir. Because the prevalence of HCV infections among patients is greatly dependent on whether they are specifically provided care with liver diseases, we classified the department where the NSI occurred as either a liver disease-related department (LD, approximately 130 beds) or a nonliver disease-related department (non-LD, approximately 1100 beds). The LDs included the Department of Gastroenterology, the Department of Infectious Diseases, and the Hepato-Biliary-Pancreatic Surgery and the Artificial Organ and Transplantation Surgery divisions of the Department of Surgery. All other departments were classified as non-LDs. Of note, 329,390 serum samples were tested for HCV-Abs, and 11,181 (3.4%) were positive during the study period. In the LDs and non-LDs, 4765 of 37,817 serum samples (12.6%) and 6416 of 291,573 serum samples (2.2%) were positive for HCV-Ab, respectively.

This study was approved by the Ethics Committee of The University of Tokyo (No. 2187) and conformed to the Declaration of Helsinki. Informed consent was obtained from all subjects included in this study using the opt-out informed consent procedure. Namely, on admission, all of the inpatients were provided with a document explaining the policy of The University of Tokyo Hospital on protection of personal information and handling of samples of human origin. Any patients could request for suspension of use, elimination, provision of own personal information. In addition, a document that declares an opt-out policy by which any possible patient could refuse to be included in this study was uploaded on the web page of the The University of Tokyo Hospital.

### Testing for HCV-Ab and HCV RNA and the definition of current infection status

All samples were tested for immunoglobulin G (IgG) antibodies against HCV using either a chemiluminescent immunoassay (cutoff value, ≥1.0 S/CO) or chemiluminescent enzyme immunoassay (cutoff value, ≥1.0 S/CO).

In patients with HCV-Ab-positive status, HCV RNA was analyzed by the Amplicor HCV test (Roche Diagnostics, Tokyo, Japan) or by real-time polymerase chain reaction by the COBAS TaqMan HCV test (Roche Diagnostics, Tokyo, Japan). We defined HCV RNA-positive patients as those with active HCV infection, including 1) an HCV RNA-positive result obtained at the time of the NSI; 2) an HCV RNA-positive result obtained before the NSI and no history of HCV treatment until the NSI; and 3) an HCV RNA-positive result obtained after the NSI and with no other possible route of HCV infection after the NSI.

### Follow up of health care workers after NSIs

We monitored HCWs after NSIs from HCV-Ab-positive patients as follows. We checked serum titers of HCV-Ab and values of liver enzymes of suffered HCWs immediately after the NSI and also after 1 month, 3 months, and 6 months. We also checked the HCV RNA of the HCW at 1 month. If at least one of the titers of HCV-Ab after 1, 3, or 6 months was above the cut-off level and/or the HCV RNA at 1 month was positive, we regarded the NSI as a cause of HCV transmission.

### Statistics

Data processing and analysis were performed with JMP Pro 15 software (SAS Institute Japan, Tokyo, Japan). Continuous variables were compared using Welch’s *t*-test or Wilcoxon’s rank-sum test, and categorical variables were compared using Fisher’s exact test. The Cochran–Armitage trend test was used to evaluate increasing or decreasing trends. A *p-*value < 0.05 was considered to indicate statistical significance.

## Results

Between 2009 and 2019, 1435 NSIs occurred. All sources of NSIs were patients, and some patients were sources of multiple NSIs. The number of total NSIs and that per 1000 beds in each year were summarized in Supplementary Table 1. Between January 2009 and December 2014 (the era before IFN-free DAAs) and between January 2015 and December 2019 (the era of IFN-free DAAs), 884 and 551 NSIs occurred, respectively. The overall rate of NSIs in our hospital was lower in the era of IFN-free DAAs than in the era before DAAs (89.7 vs 120.0 NSIs/1000 beds/year; 95% confidence interval [CI], 81.7 to 97.7 vs 102.3 to 137.5; *p* = 0.0106, Wilcoxon’s rank-sum test).

**Fig. 1:**
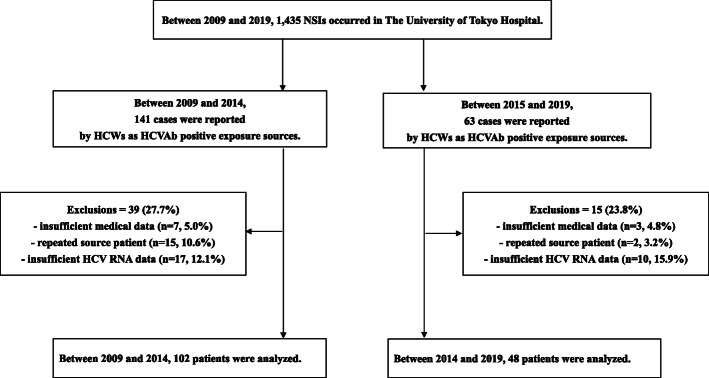
Flow diagram of needle-stick injury (NSI) case selection

Among all 1435 NSIs, 204 injuries (14.2%; 95%CI, 12.5 to 16.1%) were associated with HCV-Ab-positive source patients. Of those, 54 cases were excluded (insufficient data, *n* = 10; multiple exposures from the same patient, *n* = 17; unavailable HCV RNA viral load data, *n* = 27). The remaining 150 NSIs associated with 150 source patients were analyzed (Fig. 1). Detailed situations of 150 NSIs were as follows: incision or suture, *n* = 46; blood collection, *n* = 36; percutaneous injection, *n* = 32; catheter-related procedure, *n* = 16; body fluid or tissue collection, *n* = 5; other invasive or surgical procedures, *n* = 14; unknown, *n* = 1. Among the 150 source patients, 101 patients were HCV RNA-positive, 88 were male, and the mean age was 70.0 years old (Table 1). There was no transmission of HCV to HCWs during the entire study period.

**Table 1:** Background characteristics of the HCV-Ab-positive patients

Variables	Entire period (*n* = 150)	Between 2009 and 2014 (*n* = 102)	Between 2015 and 2019 (*n* = 48)	*p*-value
Age (years)	70.0 ± 11.8	69.9 ± 12.1	70.4 ± 11.4	0.9229
HCV RNA, positive – no. (%, 95CI)	101 (67.3, 59.5–74.3)	78 (76.5, 67.4–83.6)	23 (47.9, 34.5–61.7)	0.0007*
Gender, male – no. (%, 95CI)	88 (58.7, 50.7–66.2)	61 (59.8, 50.1–68.8)	27 (56.3, 42.3–69.3)	0.7241
Department, LDs – no. (%, 95CI)	75 (50.0, 42.1–57.9)	54 (52.9, 43.3–62.3)	21 (43.8, 30.7–57.7)	0.3816

Values are expressed as means ± SD unless otherwise stated

*HCV* hepatitis C virus, *LDs* liver disease-related departments, *CI* confidence interval

*p <  0.05

**Fig. 2:**
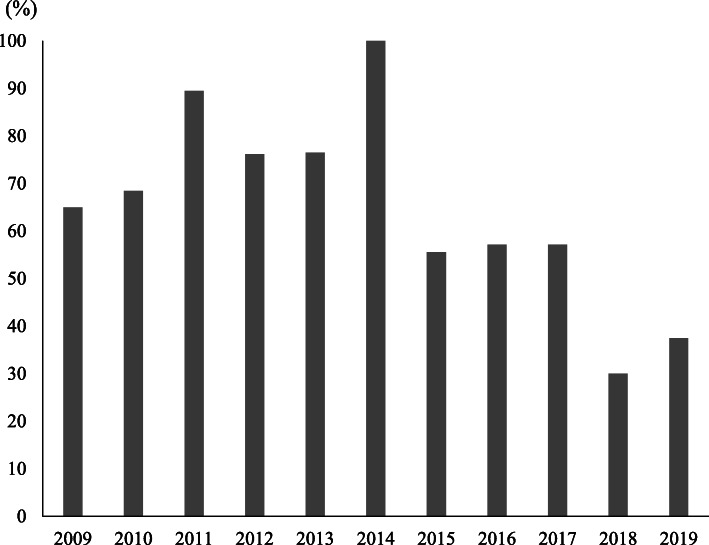
Annual transition of HCV RNA-positive rates in HCV-Ab-positive NSI source patients. The proportion of HCV RNA-positive patients among HCV-Ab-positive NSI source patients significantly changed from 2009 through 2019 (*p* = 0.005, Cochran–Armitage test)

The proportion of HCV RNA-positive patients significantly changed from 2009 through 2019 (Fig. 2, *p* = 0.005, Cochran–Armitage test). The proportion of HCV RNA-positive patients decreased from 65.0% in 2009 to 37.5% in 2019. The number of HCV-Ab-positive, HCV RNA-positive, and HCV RNA-negative NSIs in each year were summarized in Supplementary Table 1.

We then compared the source patients in the era before DAAs with those in the era of IFN-free DAAs. In the era before IFN-free DAAs 102 patients were HCV-Ab positive, and HCV RNA was analyzed. Seventy-eight of the patients were positive for HCV RNA (76.5%; 95%CI, 67.4–83.6%). On the other hand, in the era of IFN-free DAAs, 48 patients were HCV-Ab positive, and HCV RNA was analyzed. Twenty-three of the patients were positive for HCV RNA (47.9%; 95%CI, 34.5–61.7%; *p* = 0.0007 compared with 2009–2014) (Table 1).

Age, sex, and department where the NSI occurred were not significantly different between the eras before and after DAAs in the HCV-Ab-positive source patients (Table 1). However, the mean age was significantly higher in HCV RNA-positive patients than in HCV RNA-negative patients in the era before DAAs (Table 2). HCV-Ab titers were significantly higher in HCV RNA-positive patients than in HCV RNA-negative patients in both eras (Table 2).

**Table 2:** Background characteristics according to HCV RNA positivity

Variables	Between 2009 and 2014 (*n* = 102)			Between 2015 and 2019 (*n* = 48)		
	HCV-RNA Positive (*n* = 78)	HCV-RNA Negative (*n* = 24)	*p*-value	HCV-RNA Positive (*n* = 23)	HCV-RNA Negative (*n* = 25)	*p*-value
Age (years)	71.7 ± 10.6	63.8 ± 14.5	0.0122*	71.4 ± 8.8	69.4 ± 13.5	0.8123
Gender, male – no. (%, 95CI)	44 (56.4, 45.4–66.9)	17 (70.8, 50.8–85.1)	0.2412	11 (47.8, 29.2–67.0)	16 (64.0, 44.5–79.8)	0.3830
HCV-Ab titer^a^	9.8 ± 0.6	6.9 ± 2.9	< 0.0001*	10.0 ± 0.1	7.3 ± 3.4	0.0006*
Department, LDs – no. (%, 95CI)	46 (59.0, 47.9–69.2)	8 (33.3, 18.0–53.3)	0.0358*	14 (60.9, 40.8–77.8)	7 (28.0, 14.3–47.6)	0.0405*
Antiviral therapy^b^						
-Interferon, Yes – no. (%, 95CI)	27 in 75 (36.0, 26.1–47.3)	12 in 21 (57.1, 36.5–75.5)	0.1302	7 in 22 (31.8, 16.4–52.7)	10 in 23 (43.5, 25.6–63.2)	0.5420
-DAAs, Yes – no. (%, 95CI)	–	–	–	2 in 22 (9.1, 2.5–27.8)	9 in 23 (39.1, 22.2–59.2)	0.0351*

Values are expressed as means ± SD unless otherwise stated

*HCV* hepatitis C virus, *LDs* liver disease-related departments, *CI* confidence interval

^a^About HCV-Ab titer, between 2009 and 2014, 1 HCV RNA-negative patient had no result documented in the medical record

^b^About histories of antiviral therapy, between 2009 and 2014, 3 HCV RNA-positive and HCV RNA-negative patients each had no treatment history documented in the medical record. Between 2015 and 2019, 1 HCV RNA-positive and 2 HCV RNA-negative patients had no treatment history documented in their medical record. In the era after DAAs, 2 HCV RNA-positive and HCV RNA-negative patients each were treated with both IFN-based therapies and DAAs. Total number of each column was provided if there was missing data

*p < 0.05

In the era before DAAs, the rates of IFN-based therapies were not significantly different between HCV RNA-positive and HCV RNA-negative patients (Table 2). In the era of IFN-free DAAs, the rates of IFN-based therapies were also not significantly different between HCV RNA-positive and HCV RNA-negative patients. On the other hand, IFN-free DAAs therapies were significantly conducted in HCV RNA-negative patients compared with HCV RNA-positive patients. Details of the IFN-free DAAs were as follows: LDV/SOF in two HCV RNA-negative patients and two HCV RNA-positive patients, DCV/ASV in four HCV RNA-negative patients, and SOF/RBV in three HCV RNA-negative patients. There was no patient treated with the combination regimens with interferon and DAAs such as telaprevir or simeprevir.

Regarding the departments where NSIs occurred, the proportion of HCV RNA-positive patients was higher in LDs than in non-LDs, and that of HCV RNA-negative patients was higher in non-LDs than in LDs in both eras (Table 2). Particularly, the proportion of HCV RNA-positive patients was significantly lower in the era of IFN-free DAAs than in the era before DAAs in non-LDs (from 66.7% in the era before DAAs to 33.3% in the era of IFN-free DAAs, *p* = 0.0078, Table 3).

**Table 3:** Background characteristics according to department where NSIs occurred

Variables	Liver disease-related departments (*n* = 75)			Nonliver disease-related departments (*n* = 75)		
	Between 2009 and 2014 (*n* = 54)	Between 2015 and 2019 (*n* = 21)	*p*-value	Between 2009 and 2014 (*n* = 48)	Between 2015 and 2019 (*n* = 27)	*p*-value
Age (years)	70.3 ± 11.6	71.3 ± 9.2	0.9812	69.3 ± 12.7	69.7 ± 13.0	0.8424
Gender, male – no. (%, 95CI)	35 (64.8, 51.5–76.2)	12 (57.1, 36.5–75.5)	0.5998	26 (54.2, 40.3–67.4)	15 (55.6, 37.3–72.4)	1.0000
HCV RNA, positive – no. (%, 95CI)	46 (85.2, 73.4–92.3)	14 (66.7, 45.4–82.8)	0.1064	32 (66.7, 52.5–78.3)	9 (33.3, 18.6–52.2)	0.0078*

Values are expressed as means ± SD unless otherwise stated

*HCV* hepatitis C virus, *CI* confidence interval

*p < 0.05

## Discussion

In this study, the overall incidence of NSIs in our hospital was lower in the era of IFN-free DAAs than in the era before IFN-free DAAs. Continuous and accumulating education to prevent the iatrogenic infection for HCWs, including the recommendation to use safety equipment, started from 2000’s in our hospital, might contribute to the decrease in NSI cases.

Remarkable advances have been made in the treatment of chronic hepatitis C, as DAAs can efficiently eliminate HCV and patients can easily achieve an SVR [17]. We speculated that the rate of HCV-infected source patients in the era of IFN-free DAAs would be lower than that in the era before IFN-free DAAs. As expected, the proportion of HCV RNA-positive patients significantly changed from 2009 through 2019, and a significant difference was observed between the eras before and after DAAs.

To assess the impact of antiviral treatments, including IFNs and DAAs, we further analyzed the antiviral HCV treatment histories in the source patients. As expected, in the era of IFN-free DAAs, HCV RNA-negative patients had a significantly higher frequency of DAA treatment history than HCV RNA-positive patients. IFN-based therapies were also administered in HCV RNA-negative patients as well as in HCV RNA-positive patients in both the eras before and of IFN-free DAAs. Considering these results, we assume that IFN-free DAAs might have contributed to the increase in NSIs associated with HCV RNA-negative source patients between 2015 and 2019.

Regarding the departments where NSIs occurred, the proportion of HCV RNA-positive patients was higher in LDs, and that of HCV RNA-negative patients was higher in non-LDs both in the eras before and after DAAs. These results were reasonable considering the patients’ backgrounds. Namely, patients with active liver disorders, such as hepatitis C, liver cirrhosis, and hepatocellular carcinoma, were treated in LDs. In particular, the proportion of HCV RNA-positive patients was significantly decreased in the era of IFN-free DAAs compared with the era before DAAs in the non-LD group. We recommend that HCV RNA should be analyzed when HCWs detect HCV-Ab positivity in patients, particularly those treated in non-LDs; this might lead to adequate risk assessment and a reduction in the mental burdens of HCWs. The HCV-Ab titer in a patient might support the estimation of HCV RNA positivity. It was reported that HCV-Ab was still positive but its titer was continuously getting decreased for long duration after the achievement of SVR in HCV infected patients [18, 19]. Actually, in this study, the titers of HCV-Ab in HCV RNA-positive patients were approximately 10 and over, whereas those in HCV RNA-negative patients were significantly lower than 10. Therefore, HCV RNA negativity should be confirmed, especially in patients with relatively low HCV-Ab titers.

We must acknowledge some limitations of this study. First, this was a single-center cross-sectional study, and the findings need to be validated in other populations. In this study, we analyzed the specific population such as source patients of NSIs in a tertiary care hospital in Japan. Actually, HCV-Ab positive rates were different among the general population in Japan (0.63%) [13], all of the patients whose serum HCV-Ab was examined in our hospital in the same period with this study (3.4%, 11,181 in 329,390 samples), and the source patients of NSIs in this study (14.2%, 204 in 1435 NSIs), chiefly due to the differences in the background of the analyzed subjects. However, to the best of our knowledge, there was no study reporting the change in the actual HCV RNA positivity rate among HCV-Ab-positive source patients of NSIs between the eras before and after the emergence of IFN-free DAAs. In addition, the hospital in this study is one of the largest hospitals in Japan. Considering these points, the data derived in this study are vital for understanding the current situation of and planning strategies to manage NSIs. Second, some cases might have been excluded due to a lack of HCV RNA data, despite HCV RNA positivity. As a result, it is possible that we slightly underestimated the actual rate of HCV infection. However, the rate of actual HCV infection among HCV-Ab-positive cases in the era before IFN-free DAAs in this study (76.5%; 95% CI, 67.4 to 83.6%) did not differ from the rate obtained from the anti-HCV-positive population-based cohort further confirmed with a recombinant immunoblot assay for HCV in the National Health and Nutrition Examination Study during a similar period between 2007 and 2012 (77.7%; 95% CI, 72.4 to 82.2%) [10]. These results were tended to be lower than those obtained in Japan in 1992 (89.0%; 95% CI, 80.9 to 93.9%) [12]. It would be better to refer to results obtained in the era of IFN-free DAAs in Japan; however, to the best of our knowledge, no current reports exist. Notably, Japan was the first country to introduce IFN-free DAA regimens for HCV therapy. This is the first report describing changes in the HCV burden, with a focus on NSIs occurring before and after the DAA era in a hospital setting.

## Conclusions

Actual HCV RNA positivity in NSI source patients decreased after the emergence of DAAs. DAAs might have contributed to this reduction, and HCV RNA-negative patients were predominant in non-LDs in the era of IFN-free DAAs. HCV RNA should be analyzed when HCWs detect HCV-Ab positivity in hospital patients.

## Supplementary Information

**Additional file 1: Table S1.** The number of NSIs in each year.

## Data Availability

All data generated or analyzed during this study are included in this published article.

## Abbreviations

HCW: Healthcare worker
NSI: Needle-stick injury
HCV: Hepatitis C virus
HBV: Hepatitis B virus
HIV: Human immunodeficiency virus
PEP: Postexposure prophylaxis
DAAs: Direct-acting antivirals
HCV-Ab: HCV antibody
IFN: Interferon
SVR: Sustained virological response
DCV/ASV: Daclatasvir hydrochloride and asunaprevir
LDV/SOF: Ledipasvir/sofosbuvir
SOF/RBV: Sofosbuvir/ribavirin
LD: Liver disease-related department
Non-LD: Nonliver disease-related department
